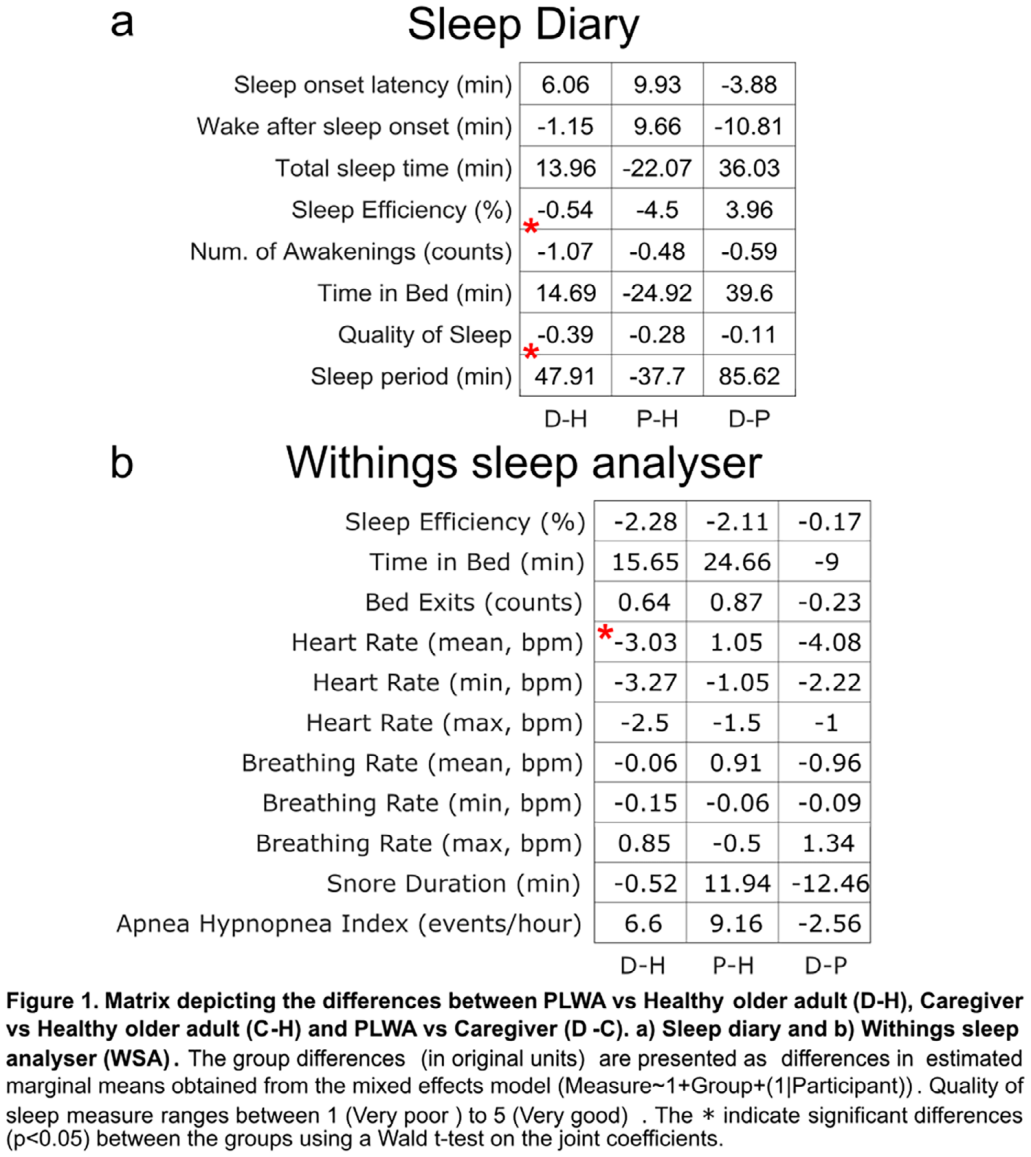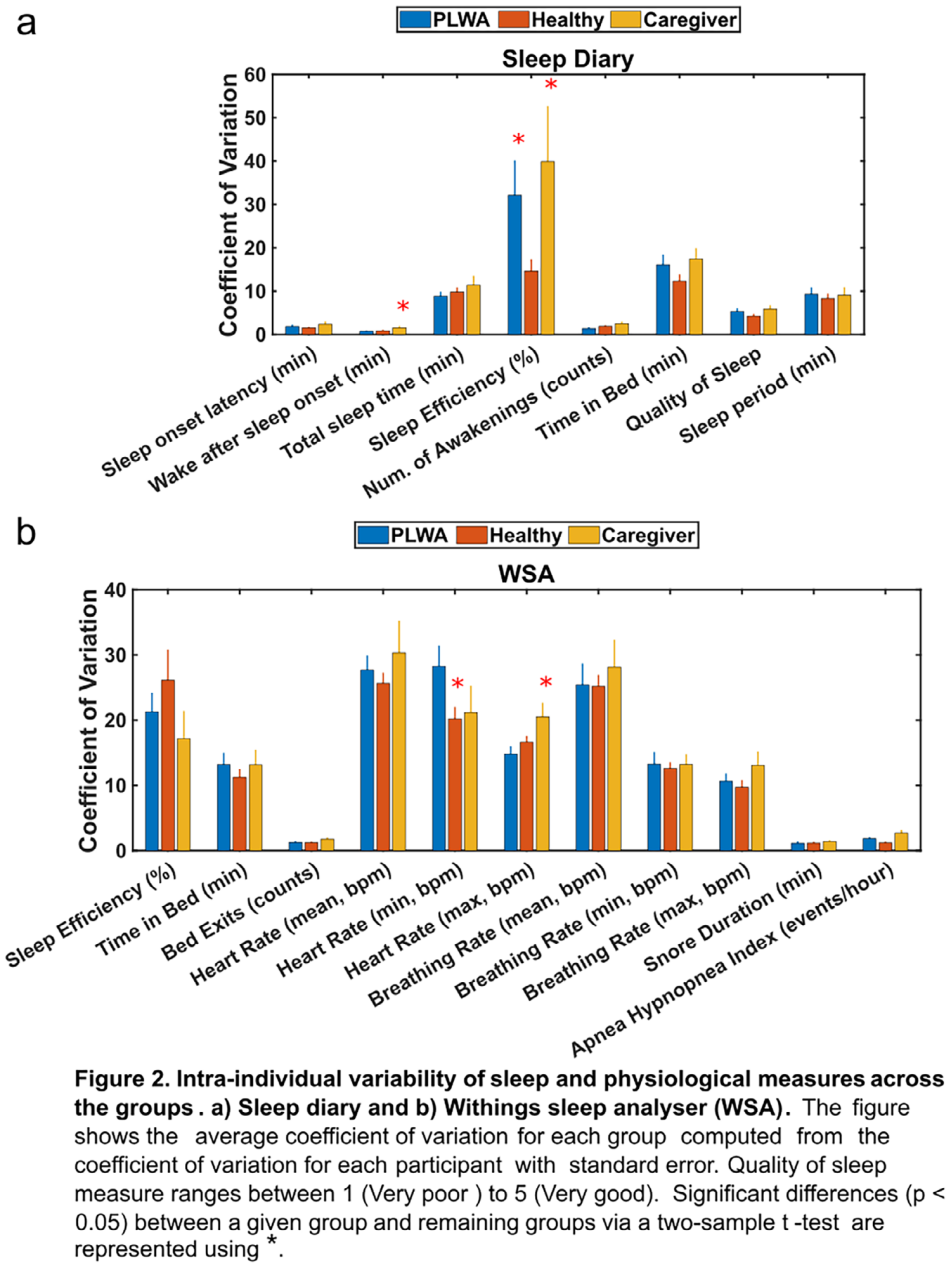# Night‐to‐Night Variation of Sleep and Heart Rate in People with Mild Alzheimer’s and Their Caregivers: Preliminary Findings from a Sleep Diary and Contactless Physiological Monitoring at Home

**DOI:** 10.1002/alz.092492

**Published:** 2025-01-09

**Authors:** Kiran K.G. Ravindran, Ciro della Monica, Giuseppe Atzori, Hana Hassanin, Ramin Nilforooshan, Victoria Louise Revell, Derk‐Jan Dijk

**Affiliations:** ^1^ UK Dementia Research Institute, Care Research and Technology Centre, Imperial College London and the University of Surrey, Guildford United Kingdom; ^2^ Surrey Sleep Research Centre (SSRC), University of Surrey, Guildford, Surrey United Kingdom; ^3^ National Institute for Health Research ‐ Royal Surrey Clinical Research Facility, Guildford, United Kingdom, Guildford, Surrey United Kingdom; ^4^ Surrey Clinical Research Facility, University of Surrey, Guildford, Surrey United Kingdom; ^5^ Surrey and Borders Partnership NHS Foundation Trust, Chertsey United Kingdom

## Abstract

**Background:**

Changes in sleep patterns are common in Alzheimer’s disease and impact the quality of life of both people living with Alzheimer’s (PLWA) and their caregivers. Longitudinal recordings and assessment of night‐to‐night variations in sleep and physiology can improve our understanding of how sleep influences clinical outcomes and caregiver wellbeing.

**Method:**

We collected sleep diary and contactless sleep technology data (Withings sleep analyser, WSA) in community dwelling PLWA (N = 16, Age = 72.8 ± 5.7 years, SMMSE = 27.1 ± 1.4), caregivers (N = 8, Age = 74.4 ± 3.8 years, SMMSE = 28.6 ± 0.7) and healthy older adults (N = 23, Age = 70.7 ± 5.5 years, SMMSE = 29.0 ± 0.9) for a period of upto 14 days at home. Group differences were analysed using a mixed model with participants as random effect while coefficients of variation were used to quantify the intra‐individual variability.

**Result:**

Sleep diary and contactless sleep and heart rate variables were successfully collected for 624 (sleep diary) and 647 (WSA) nights. Analyses of group means of the sleep diary measures revealed that the self‐reported sleep period was significantly longer (p<0.001) in PLWA compared to healthy older adults. The number of reported night awakenings was larger (p = 0.019) in caregivers compared to PLWA. The group mean of nightly minimum heart rate estimated by WSA was significantly lower (p = 0.015) in PLWA compared to healthy older adults (Figure 1).

In addition to these differences in group averages, we also observed differences in the night‐to‐night variability of sleep and heart rate measures. The variability of wake after sleep onset and sleep efficiency as reported in the sleep diary were larger in caregivers compared to PLWA and healthy older adults (p<0.05) (Figure 2). The night‐to‐night variability of maximum heart rate was larger in caregivers while the variability of minimum heart rate was larger in PLWA, and caregivers compared to healthy older adults (p<0.05).

**Conclusion:**

These data indicated that longitudinal nocturnal recordings of sleep and physiology and analyses of night‐to‐night variability provide insights into the extent of disturbances of sleep and physiology in PLWA and their caregivers.